# Exercise therapy after ultrasound-guided corticosteroid injections in patients with subacromial pain syndrome: a randomized controlled trial

**DOI:** 10.1186/s13075-016-1002-5

**Published:** 2016-06-04

**Authors:** Karen Ellegaard, Robin Christensen, Sara Rosager, Cecilie Bartholdy, Søren Torp-Pedersen, Thomas Bandholm, Bente Danneskiold-Samsøe, Henning Bliddal, Marius Henriksen

**Affiliations:** The Parker Institute, Copenhagen University Hospital, Bispebjerg og Frederiksberg, Nordre Fasanvej 57, DK-2000 Copenhagen F, Denmark; Physical Medicine & Rehabilitation Research – Copenhagen [PMR-C], Department of Physical Therapy, Hvidovre Hospital, University of Copenhagen, Copenhagen, Denmark; Clinical Research Centre, Hvidovre Hospital, University of Copenhagen, Copenhagen, Denmark; Department of Orthopedic Surgery, Hvidovre Hospital, University of Copenhagen, Copenhagen, Denmark; Department of Radiology, Rigshospitalet Glostrup, University of Copenhagen, Copenhagen, Denmark; Centre for Sensory-Motor Interaction, Aalborg University, Aalborg, Denmark; Department of Physical and Occupational Therapy, Copenhagen University Hospital, Bispebjerg og Frederiksberg, Copenhagen, Denmark

**Keywords:** Subacromial pain syndrome, Glucocorticosteroid injection, Exercise therapy, Ultrasound imaging, Randomized controlled trial

## Abstract

**Background:**

Subacromial pain syndrome (SAPS) accounts for around 50 % of all cases of shoulder pain. The most commonly used treatments are glucocorticosteroid (steroid) injections and exercise therapy; however, despite treatment SAPS patients often experience relapse of their symptoms. Therefore the clinical effect of combining steroid and exercise therapy is highly relevant to clarify. The aim of this randomized controlled trial was to investigate if exercise therapy added to steroid injection in patients with SAPS will improve the effect of the injection therapy on shoulder pain.

**Methods:**

In this two-arm randomized trial running over 26 weeks, patients with unilateral shoulder pain (> 4 weeks) and thickened subacromial bursa (> 2 mm on US) were included. At baseline all participants received two steroid injections into the painful shoulder with an interval of one week. Subsequently they were randomized (1:1) to either 10 weeks exercise of the involved shoulder (intervention group) or exercise of the uninvolved shoulder (control group). The patients were re-examined after the exercise program (at week 13) and again at week 26. The primary outcome assessed after 26 weeks was change in shoulder pain analyzed using the intention-to-treat principle (non-responder imputation).

**Results:**

Ninety-nine SAPS patients (58 female) participated (49 intervention/50 control). At both follow up visits (week 13 and 26) no statistically significant between-group differences in pain changes on a visual analog scale (mm) were seen (13 weeks: pain at rest 1.7 (95 % CI –3.6 to 7.0; *P* = 0.53); pain in activity 2.2 (95 % CI –6.5 to 10.9; *P* = 0.61), 26 weeks: rest 5.6 (95 % CI –0.9 to 12.1; *P* = 0.09); activity 2.2 (95 % CI –6.8 to 11.2; *P* = 0.62). The reduction in pain was most evident in the control group at all four pain measurements. The only difference between groups was seen by US examination at week 13, where fewer participants with impingement were observed in the intervention group compared with the controls (9 vs. 19 participants; *P* = 0.03).

**Conclusion:**

Exercise therapy in the painful shoulder in SAPS patients did not improve the effectiveness of steroid injections for shoulder pain in patients with unilateral SAPS and enlarged subacromial bursa on US examination.

**Trial registration:**

ClinicalTrials.gov (NCT01506804). Registration date 5 May 2011.

**Electronic supplementary material:**

The online version of this article (doi:10.1186/s13075-016-1002-5) contains supplementary material, which is available to authorized users.

## Background

Shoulder pain is common and accounts for around 12 % of the contacts in primary care [1, 2] with subacromial pain syndrome (SAPS) accounting for approximately 50 % of cases of shoulder pain [3, 4]. SAPS is suggested to be a more comprehensive diagnosis than subacromial impingement to describe unspecific shoulder pain. The SAPS definition can be used as an umbrella diagnosis for all conditions affecting the structures of the subacromial space including enlarged subacromial bursa, rotator cuff and biceps tendon ruptures, and other pathological changes in the tendons [3, 5, 6].

The most commonly used treatments for SAPS are glucocorticosteroid (steroid) injection and exercise therapy. SAPS is often recurrent, so patients often receive an unstructured mix of both treatments [3, 7–10]. Effectiveness of each treatment has been demonstrated in the short term on pain and function. The choice of treatment is ambiguous and there is no standardized treatment algorithm, and the long-term effects of a stringent protocol investigating steroid injection and exercise therapy in combination have not been investigated [3, 7–10].

Inflammation and pain may impair muscle function [11]. Experimental studies have shown that pain alters muscle recruitment strategies and strength [12–14]. Accordingly, it is plausible that pharmacological suppression of inflammation and pain prior to exercise may enhance the effect of the exercise. Also, exercise may suppress the inflammation [15] and may per se have additional effects on the pharmacological treatment of the inflammation [16]. The aim of the current study was to test whether the effect of steroid injections on SAPS may be improved by adding exercise therapy.

## Methods

We conducted an assessor-blinded, two-arm, parallel-group, randomized controlled trial running over 26 weeks; primary outcome was assessed after 26 weeks (primary end point). A methodological protocol was developed and registered with ClinicalTrials.gov before the trial began (NCT01506804) 05/05-2011. The protocol was also approved by the Regional Health Research Ethics Committee (H-4-2010-022). The trial was conducted in accordance with the Helsinki Declaration and this paper follows the CONSORT reporting guidelines [17]. All participants signed an informed consent form before participating in the study.

### Setting and eligibility criteria

Participants were recruited from a rheumatology outpatient clinic at a Hospital in Denmark and by advertising on a specific site on the Internet for participants for clinical trials, between May 2009 and April 2011.

Inclusion criteria were age 18 to 70 years and unilateral shoulder pain provoked by active shoulder abduction and lasting at least 4 weeks. In addition, an enlarged subacromial bursa (≥ 2 mm) as assessed by ultrasound imaging (US) in the symptomatic shoulder was required.

Exclusion criteria included complete or partial ruptures of the biceps or rotator cuff tendons assessed by US [18], symptoms originating from the cervical spine, other conditions explaining the shoulder pain, capsular pattern restriction, contraindications to steroid or lidocaine or exercise therapy, steroid injection of either shoulder within the previous 3 months, and any previous shoulder surgery.

### Procedures

Potentially eligible participants having received oral and written information about the study were invited to a clinical examination by a rheumatologist and an US examination of the shoulder. Upon inclusion, and subsequent to baseline assessments, the participants received two US-guided injections of steroid in the thickened bursa in the painful shoulder, given with an interval of 1 week. Two injections were given to increase the chances of a clinical effect. After the second injection, the participants were randomized. The intervention group received a training program for the painful shoulder. The control group received the same program for the asymptomatic shoulder. Both groups commenced the training program 1 week after receiving the second injection. The exercise program lasted for 10 weeks.

### Randomization, treatment allocation and blinding

The participants were randomized 1:1 in blocks of 4–6 using an envelope-based lottery. The individual allocations were held in sealed opaque envelopes. The leader of the project (KE), who was blinded to all clinical data, made the allocation. Due to the type of treatment in both the intervention and control group neither the participant nor the person performing the intervention were blinded. The persons performing the clinical tests and training sessions in both intervention and control group were blinded to the self-reported shoulder function. The person performing all the statistical analysis was blinded to group allocation.

### Interventions

#### Injections

The two injections given (separated by 1 week) each contained 1 mL methylprednisolone (40 mg Depo-Medrol®, Pfizer) and 2 mL lidocaine (5 %). All injections were given US-guided, as it has been shown that the effect on pain and function is superior to blind injections [19–21]. An experienced radiologist specializing in US (STP) gave the injections. The US examinations were carried out using a Logic E9 with a 15 MHz linear array transducer (General Electric Medical System, Milwaukee, WI, USA).

Patients sat in an upright position. The arm was positioned behind the lower back, with the elbow flexed. The US probe was placed on the lateral contour of the shoulder parallel to the underlying supraspinatus tendon. The injection was performed with a lateral approach and real-time US evidence of the bolus being correctly distributed in the enlarged bursa.

#### Exercise

The exercise intervention followed the descriptors by Toigo & Boutellier [22]. The exercise program consisted of three sessions per week for 10 weeks, with one session per week being supervised by a physiotherapist; the other was home based. Additional file 1 describes the exercise program in detail. If the exercises caused shoulder pain rated as > 50 mm on a 10–mm visual analog scale (VAS), which did not subside immediately, loading was reduced. A sensation of post exercise muscle fatigue and delayed onset muscle soreness was regarded as acceptable. The same exercise program was used in both groups, but on different shoulders (involved/non-involved as per the allocation).

During the first 2 weeks of exercise, (phase 1), the emphasis was on scapula muscle control and strength. The following 8 weeks (phase 2) aimed at progressive strengthening of the muscles of the rotator cuff. The program rationale was to target muscle function deficits of the scapula prior to targeting muscle function deficits of the rotator cuff muscles [4, 23]. An exercise diary was used in both groups.

### Outcome measurements and assessment

Outcomes were measured at baseline (before the first injection), at the week-13 visit (after the exercise program), and at a 26-week follow-up visit (12 weeks after cessation of the exercise program). As the purpose of this study was to assess the effect of stringent combined pharmacological and exercise therapy the co-primary outcomes were changes from baseline in current shoulder pain during active shoulder abduction and at rest at the 26-week follow up (primary end point). These pain measures were assessed on 0–100 mm VAS with anchors being 0 = no pain and 100 = worst imaginable pain. The secondary outcomes were the same pain ratings at week 13; clinical impingement signs (Hawkins-Kennedy test), US impingement test, subacromial bursa size on US (size; mm), self-reported shoulder function (Shoulder Disability Questionnaire (SDQ)), and isometric muscle strength.

#### Impingement

In the Hawkins-Kennedy sign the shoulder pain is reproduced by shoulder flexion followed by a forced internal rotation [24]. The test is positive if the shoulder pain is reproduced. On US-based impingement the acromion and the humeral head were simultaneously visualized by US during passive abduction of the shoulder joint. Impingement was indicated if the bursa and/or tendons were impinged at the edge of the acromial bone. Subacromial bursa enlargement was assessed from US examinations in two positions: (1) with the patients sitting in an upright position with the arm positioned behind the back and (2) in the same position but with the hand on the waist. In both positions, the transducer was moved until it was over the area where the thickness of the bursa was most pronounced. The maximal bursa size was obtained in both positions. We defined a bursa thickness > 2 mm as enlarged [25].

#### Isometric muscle strength

IMS was assessed unilaterally (painful shoulder) using a handheld dynamometer (Commander Power track II, JTEC Medical, USA). This method has been shown to be reliable for assessment of muscle strength in the shoulder and hip [26, 27]. IMS was measured in internal rotation, external rotation, and abduction, with each contraction lasting approximately 6 seconds. The abduction strength was tested in 45° shoulder abduction. IMS in internal and external rotation was assessed with the shoulder in the neutral position and the elbow flexed at 90°. All measurements were repeated four times with 10-second intervals and the maximum value was used for analysis. Standardized instructions and verbal encouragement were given [28].

#### Self-reported shoulder function

Self-reported functional status of the shoulder was assessed by the SDQ. The questionnaire consists of 16 items assessing pain during the previous 24 h in the shoulder during activities of daily living [29, 30]. Three answers are possible in each item: “yes”, “no” and “not applicable”. The shoulder function is measured as a score between 0 and 100 % expressing the proportion of “yes” scores in relation to the total number of answers. The “not applicable” items are not included in the analysis [31]. A Danish version of the SDQ was not available, but the English version is validated [3]. Two people with good English skills translated the test independently. The two translated versions were compared by the translators and the study manager. Any discrepancies in translations were discussed until consensus was reached.

### Sample size and power considerations

This study was powered as a superiority trial for a comparison between the participants allocated to exercise of the painful shoulder (intervention) and those allocated to exercise of the non-painful shoulder (control). Assuming that the intervention produced a reduction in one of the co-primary outcomes (pain during active shoulder abduction and pain at rest) that was 10 mm larger than the control with a standard deviation of 20 mm and a conjectured correlation of 0.7 between the covariate (baseline pain) and the response (change in pain), we calculated that we would need 42 participants per group to test a two-tailed hypothesis with 80 % power and a 2.5 % significance level (*P* < 0.025), using the usual *F* test of the group effect in the general linear univariate model with fixed class effects (group; two levels (intervention vs. control)) with an additional fixed covariate (baseline pain). To account for attrition we decided to include 100 patients in total, yielding a power of 88.8 %. Although we also investigated effects on other outcomes, we did not power the trial for this because we had no a priori assumptions about effect sizes in our secondary outcomes and thus a larger trial may be needed to reliably detect these.

### Statistical analysis

All data analyses were carried out according to a pre-established analysis plan. Analyses were done applying SAS (v. 9.2; SAS Institute Inc., Cary, NC, USA). All descriptive statistics and tests are reported in accordance with the recommendations of the Enhancing the Quality and Transparency Of health Research (EQUATOR) network, as per the Consolidated Standards of Reporting Trials (CONSORT) statement [32]. In order to evaluate the empirical distributions of continuous outcomes, visual inspection was used to suggest whether the assumption of normality was reasonable.

All patients randomized were assessed for efficacy and safety (i.e., intention-to-treat (ITT) population). At 13 weeks (the end of intervention) and after 26 weeks, the exercise and control groups were compared using analysis of covariance (ANCOVA) to analyze mean changes from baseline in the outcomes. The model included the change as the dependent variable, with treatment group as a main effect and the baseline score as an additional covariate.

The proportion of patients, who were impingement-positive on US and/or on clinical assessment at the 13-week and 26-week follow up, were analyzed using the chi-square test to evaluate the differences between proportions between the exercise and control group and the group differences are presented as risk differences with 95 % confidence interval.

The baseline observation carried forward (BOCF) approach was used for patients who did not complete the study, as this method seems conservative in self-help management programs; i.e., using a non-responder imputation. As this trial was designed as a pragmatic trial, baseline data were only imputed when the patient did not attend follow-up visits, and was not based on adherence rate or compliance considerations. Unless stated otherwise, results are expressed as the mean difference between the groups and 95 % confidence intervals (95 % CI) with the associated *P* values, based on the ANCOVA model.

## Results

**Fig. 1:**
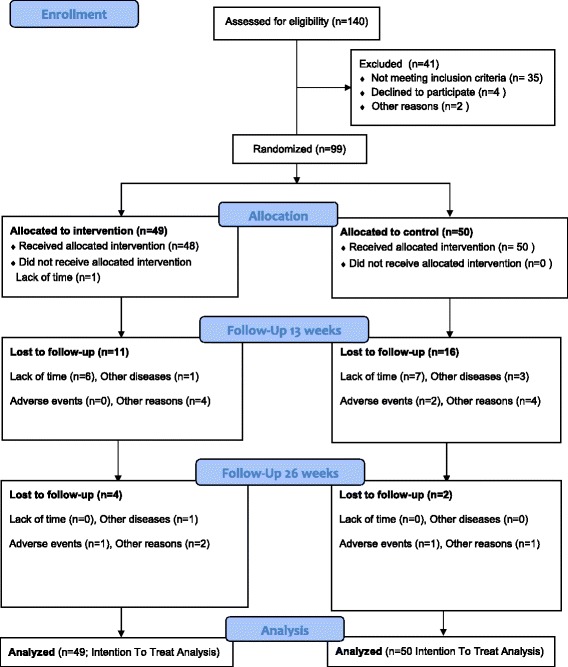
Study flow chart

The flow of patients through the trial can be seen in Fig. 1: 99 patients were included in the study. Participant number 100 volunteered but did not attend the baseline assessment and was not randomized. Because the treatment allocation was done in blocks we decided to commence the last block of participants even though the last participant was missing. Characteristics of the study sample are presented in Table 1. In the intervention group, 17 participants were lost to follow up; in the control group 18 patients were lost to follow-up (Fig. 1).

**Table 1:** Baseline characteristics for all randomized patients

	Intervention (*n* = 49) Mean (SD)	Control (*n* = 50) Mean (SD)	Combined (*n* = 99) Mean (SD)	Min	Max
Age (years)	49.4 (13.1)	47.7 (13.3)	48.5 (13.1)	18.1	69.1
Female, *n*	30 (61 %)^b^	28 (56 %)^b^	58 (59 %)^b^	-	-
Pain rest, mm VAS	5 (0 to 24)^a^	11 (0 to 30)^a^	7 (0 to 29)^a^	0	77
Pain activity, mm VAS	38 (19 to 60)^a^	48 (25 to 62)^a^	45 (23 to 62)^a^	0	100
Ultrasound variables					
Clinical impingement, yes	45 (92 %)^b^	45 (90 %)^b^	90 (91 %)^b^	-	-
Ultrasound impingement, yes	16 (34 %)^b^	25 (50 %)^b^	41 (42 %)^b^	-	-
Bursa thickness, mm	2.63 (0.59)	2.74 (0.82)	2.68 (0.71)	1.6	5.4
Shoulder Disability Questionnaire					
Percentage score in difficulty performing shoulder task, 0–100 %	65.9 (24.8)	68.4 (20.8)	67.1 (22.8)	9.1	100
Muscle strength					
MVC abduction, N	70.4 (48.4 to 103.0)^a^	63.8 (44.0 to 85.8)^a^	67.1 (46.2 to 94.6)^a^	24.2	209.0
MVC internal rotation, N	138.0 (110.0 to 184.0)^a^	118.0 (81.4to 167.0)^a^	126.0 (99.0 to 171.0)^a^	41.8	325.0
MVC external rotation, N	112.0 (85.8 to 136.0)^a^	99.0 (81.4to 129.0)^a^	104.0 (81.4 to 134.0)^a^	39.6	193.0

Data are presented as mean (SD) except where stated otherwise. *VAS* visual analog scale, *MVC* maximal voluntary contraction. ^a^Data are median (interquartile range). ^b^Data are number of participants (%)

### Compliance with exercise

Exercise diaries were only collected from the participants who completed the study (*n* = 65) and the compliance rate was calculated for these participants. In the intervention group 27 of the 33 completers filled in an exercise diary with an average number of exercise sessions of 29 out of 30 (97 %). In the control group, 31 of 32 participants filled in a diary and the number of exercise sessions was 26 out of 30 (87 %).

### Primary outcomes

**Fig. 2:**
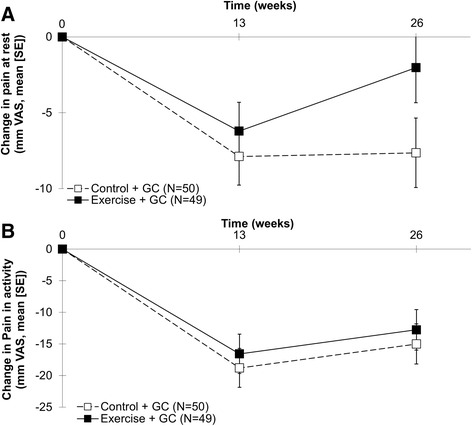
Group patterns of changes from baseline in self-reported pain at rest (**a**) and during activity (**b**). *VAS* visual analog scale, *GC* glucocorticosteroid

We found no statistically significant differences between groups in the co-primary pain outcomes at week 26 (Fig. 2). The group difference in change from baseline in pain during active abduction was 2.2 mm (95 % CI –6.8 to 11.2; *P* = 0.62). The group difference in change from baseline in pain at rest was 5.6 mm (95 % CI –0.9 to 12.1; *P* = 0.088), potentially in favor of the control group.

At the 13-week follow up the group difference in change from baseline in pain at rest was 1.7 mm (95 % CI –3.6 to 6.0; *P* = 0.53) and the group difference in change from baseline in pain during shoulder abduction was 2.2 mm (95 % CI –6.5 to 10.9; *P* = 0.61), potentially in favor of the control group.

### Secondary outcomes

There were no statistically significant differences between the groups in thickness of the subacromial bursa as seen on US, self-reported shoulder function (SDQ), clinical impingement test or isometric muscle strength at the 13-week or 26-week follow up (Table 2). At the 13-week follow up, there was a statistically significant difference between groups in the proportion of participants with a positive US impingement test, as only 18 % had a positive test in the intervention group as compared 38 % with a positive test in the control group (risk difference 0.20 (95 % CI 0.02 to 0.37); *P* = 0.03) (Table 2).

**Table 2:** Changes from baseline in secondary outcomes after the exercise program (13 weeks) and after 26 weeks Analyses were based on the Intention to Treat Population.”

	13 weeks				26 weeks			
Painful shoulder	Interv. (*n* = 49)	Cont. (*n* = 50)	Group difference	*P*	Interv. (*n* = 49)	Cont. (*n* = 50)	Group difference	*P*
	Mean (SE)	Mean (SE)	Mean (95 % CI)		Mean (SE)	Mean (SE)	Mean (95 % CI)	
Ultrasound variables								
Bursa size decrease (mm)	–0.16 (0.08)	–0.30 (0.08)	0.14 (–0.10 to 0.37	0.25	–0.16 (0.08)	–0.04 (0.08)	–0.13 (–0.36 to 0.10)	0.27
Number of positive US Impingement (positive baseline)	9 (18 %)^a^ (16 (34 %))	19 (38 %)^a^ (25(50 %))	–0.20 (–0.02 to –0.37)^b^	0.030^c^	14 (29 %)^a^ (16 (34 %))	23 (46 %)^a^ (25(50 %))	–0.17 (–0.36 to 0.01)^b^	0.073^c^
Positive clinical impingement (positive baseline)	38 (78 %)^a^ (45 (92 %))	42 (84.%)^a^ (45 (90 %))	–0.06 (–0.22 to 0.09)^b^	0.42^c^	40 (82 %)^a^ (45 (92 %))	47 (94 %)^a^ (45 (90 %))	–0.12 (–0.25 to 0.00)^b^	0.059^c^
Shoulder Disability Questionnaire								
Decrease (percentage) in difficulty performing shoulder task (0–100 %)	–26 (4)	–22 (4)	–4 (–16 to 7)	0.44	–18 (4 %)	–16 (4)	–2 (–13 to 10)	0.73
Muscle strength								
MVC abduction (*n*)	4.6 (2.5)	3.7 (2.5)	0.9 (–6.2 to 7.9)	0.80	0.5 (2.4)	1.4 (2.4)	–0.9 (–7.5 to 5.7)	0.80
MVC internal rotation (*n*)	0.8 (3.2)	3.6 (3.1)	–2.8 (–11.6 to 5.9)	0.52	0.1 (3.1)	–1.5 (3.1)	1.6 (–7.2 to 10.3)	0.71
MVC external rotation (*n*)	7.0 (2.4)	5.8 (2.4)	1.2 (–5.6 to 8.0)	0.72	–1.5 (2.2)	2.5 (2.2)	–4.1 (–10.2 to 2.1)	0.20

Analyses were based on the intention-to-treat population. *Intervent.* intervention group, *Cont.* control group, *SE* standard error, *CI* confidence interval, *US* ultrasound, *MVC* maximal voluntary contraction. ^a^Data are number of participants (%). ^b^Data are risk differences (95 % CI). ^c^Based on the Chi^2^ test of proportions

## Discussion

We investigated the effects of unilateral exercise therapy after steroid injections in the subacromial bursa in patients with SAPS. The exercise intervention was compared to exercise of the asymptomatic shoulder. There was no difference between the groups in the primary outcomes of shoulder pain at rest and activity at the 26-week follow up, although a tendency towards less pain at rest in the control group was observed. A group difference in US impingement at week 13 in favor of the intervention group was observed; however, more participants in the control group (25 vs. 16) had US impingement at baseline. This indicates that the result should be interpreted with caution. The difference was not maintained at week 26. No group differences were observed in any of the other secondary outcomes.

The equal beneficial change in pain in rest and activity in both groups is in agreement with a previous study of patients with SAPS [33]. In that study the exercise intervention was more intense, with exercise twice daily. These extensive exercises improved the shoulder function. This may indicate that any exercise program can improve shoulder pain, but intensive exercise programs are necessary in order to improve shoulder function. This is supported by a study by Bennell et al. [34] in which there was no difference between an exercise and a placebo group after 10 weeks of daily therapy. However, there was a statistically significant difference between groups 12 weeks after the end of the interventions. As no standardized intervention was offered in the follow-up period the results must be interpreted tentatively.

In contrast to other studies investigating the effect of exercise therapy we added no other physiotherapy techniques, such as manual therapy, to the training or control interventions, thus minimising the bias of a mixed intervention [33, 34].

In our study, we observed a minor increase in muscle strength in both groups, but no differences between groups. The intensity of our exercise program is comparable to the standard care offered to patients with SAPS in clinical practice [8, 9, 35–37]. Furthermore, our exercise intervention is in accordance with the newest clinical guideline for SAPS, in which US-guided steroid injections are also recommended as a part of the management of SAPS [3]. There is no consensus on the number of steroid injections needed in patients with SAPS, thus our choice of two injections was pragmatic and based on our clinical experience. One might argue that despite a rather intensive treatment, the changes in all assessed parameters from baseline were minor in both groups. This may underline the fact that there is no highly effective treatment for patients with SAPS and enlarged bursa.

Another study comparing exercise therapy after steroid injection with exercise therapy alone [35] found no difference between the groups in pain and function. Even though that study investigated steroid injection as an add-on to exercise, as opposed to our study, the results of both studies indicate that a combination of the two treatment strategies does not improve the treatment outcome in SAPS. This suggests that the choice of injection, exercise, or their combination, must depend on an individual assessment including patient and physician preferences.

Patients with SAPS form a heterogeneous group and clinical tests used to verify specific diagnoses and guide treatment are not particularly reliable or valid [6, 38]. It has been highlighted that the uncertain clinical classification may affect the outcomes of clinical studies [35]. In an attempt to overcome this problem, the participants in our study were included based on subacromial bursa enlargement identified on US. To our knowledge this is the first study using a standardized US examination to define the study population in SAPS. There is no consensus on the threshold to define pathological thickening of the subacromial bursa, but the majority of the literature applies a bursa thickness of < 2 mm as normal [25, 39, 40]. Albeit arbitrary, we used this threshold as an inclusion criterion and our study population can be considered as having SAPS with concomitant US-based pathological change. We also used US to rule out any tendon tear of the rotator cuff and biceps tendon. The use of US as the first choice imaging modality in patients with SAPS is in agreement with the newest guidelines [3].

Our cohort reflected the general population of persons with shoulder complaints as the mean age was around 50 years and the majority was women [1, 2]. As explained to the control group, the intervention on the contralateral shoulder in our study may indeed be suspected of inducing a cross-over effect. With respect to muscle strength, gains in the untrained extremity have been reported to account for about one third of the improved strength in the trained extremity [30]. This cross-over might be a limitation of our study and in support of a cross-over effect, our results indicated that the control group increased their external shoulder rotation muscle strength in the untrained, painful arm. However, the absence of increased muscle strength in abduction and internal rotation in both groups to some extent contradicts this bias. Similarly, an effect of the control exercise on pain is certainly possible. A study has shown improvements in knee pain following isolated hip abductor strengthening exercises [41]. This consideration also suggests that contralateral or general exercises can be used in cases where exercises of the involved shoulder cannot be accomplished. The reason for choosing training of the contralateral shoulder as control, despite the described effects of this training, was owed to the well-known positive effect of attention from a health professional. In order to avoid this bias, we decided that the persons in both groups should have exactly the same attention from a physiotherapist. Further, the additional value of exercise therapy might be less pronounced in the dominant arm, but this was not recorded and therefore cannot be explored.

It is a limitation of our study that the exercise compliance was only evaluated in the completer participants, and it is not unlikely that the participants lost to follow up had poor adherence to the programs. Nevertheless, the compliance was comparable between groups, which indicates that bias due to difference in exercise compliance was small in our study. Another limitation of the study is that the attrition rate was relatively high. The most common reason for drop-out was lack of time. The reported effect of the steroid injections among drop-out participants was comparable to the compliant subjects (*n* = 12 vs. *n* = 16; missing data, *n* = 6), indicating that the initial effect of the study participation was not the main reason for leaving the study.

## Conclusions

In patients with SAPS and enlarged subacromial bursa, 10 weeks of unilateral exercise of the symptomatic shoulder, given as an add-on to two sequential steroid injections into the subacromial bursa, did not improve the primary outcome of shoulder pain, compared to exercise of the asymptomatic shoulder (control). Effects on functional ability, clinical impingement signs, or muscle strength when compared to exercise of the uninvolved shoulder were also comparable between groups. The results indicate a short-term benefit on US-assessed impingement test immediately after the exercise program; however, this effect was not maintained at the 26-week follow up.

## Additional file

Additional file 1: The exercise intervention. The file describes the exercise intervention in detail. (DOC 35 kb)

## Abbreviations

ANCOVA: analysis of covariance
BOCF: baseline observation carried forward
EQUATOR: Enhancing the Quality and Transparency Of health Research
IMS: isometric muscle strength
SAPS: subacromial pain syndrome
SDQ: Shoulder Disability Questionnaire
US: ultrasound imaging
VAS: visual analog scale

## References

[1] Greving K, Dorrestijn O, Winters JC, Groenhof F, van der Meer K, Stevens M (2012). Incidence, prevalence, and consultation rates of shoulder complaints in general practice. Scand J Rheumatol.

[2] van der Windt DA, Koes BW, de Jong BA, Bouter LM (1995). Shoulder disorders in general practice: incidence, patient characteristics, and management. Ann Rheum Dis.

[3] Diercks R, Bron C, Dorrestijn O, Meskers C, Naber R, de Ruiter T (2014). Guideline for diagnosis and treatment of subacromial pain syndrome. Acta Orthop.

[4] Michener LA, McClure PW, Karduna AR (2003). Anatomical and biomechanical mechanisms of subacromial impingement syndrome. Clin Biomech (Bristol, Avon).

[5] Cools AM, Cambier D, Witvrouw EE (2008). Screening the athlete’s shoulder for impingement symptoms: a clinical reasoning algorithm for early detection of shoulder pathology. Br J Sports Med.

[6] Kelly SM, Brittle N, Allen GM (2010). The value of physical tests for subacromial impingement syndrome: a study of diagnostic accuracy. Clin Rehabil.

[7] Buchbinder R, Green S, Youd JM (2003). Corticosteroid injections for shoulder pain. Cochrane Database Syst Rev..

[8] Green S, Buchbinder R, Hetrick S (2003). Physiotherapy interventions for shoulder pain. Cochrane Database Syst Rev..

[9] Hanratty CE, McVeigh JG, Kerr DP, Basford JR, Finch MB, Pendleton A (2012). The effectiveness of physiotherapy exercises in subacromial impingement syndrome: a systematic review and meta-analysis. Semin Arthritis Rheum.

[10] Koester MC, Dunn WR, Kuhn JE, Spindler KP (2007). The efficacy of subacromial corticosteroid injection in the treatment of rotator cuff disease: A systematic review. J Am Acad Orthop Surg.

[11] Rice DA, McNair PJ (2010). Quadriceps arthrogenic muscle inhibition: neural mechanisms and treatment perspectives. Semin Arthritis Rheum.

[12] Bandholm T, Rasmussen L, Aagaard P, Diederichsen L, Jensen BR (2008). Effects of experimental muscle pain on shoulder-abduction force steadiness and muscle activity in healthy subjects. Eur J Appl Physiol.

[13] Henriksen M, Alkjaer T, Lund H, Simonsen EB, Graven-Nielsen T, Danneskiold-Samsoe B (2007). Experimental quadriceps muscle pain impairs knee joint control during walking. J Appl Physiol.

[14] Henriksen M, Rosager S, Aaboe J, Graven-Nielsen T, Bliddal H (2011). Experimental knee pain reduces muscle strength. J Pain.

[15] Petersen AM, Pedersen BK (2005). The anti-inflammatory effect of exercise. J Appl Physiol.

[16] Hagen KB, Dagfinrud H, Moe RH, Osteras N, Kjeken I, Grotle M (2012). Exercise therapy for bone and muscle health: an overview of systematic reviews. BMC Med..

[17] Schulz KF, Altman DG, Moher D (2011). CONSORT 2010 statement: updated guidelines for reporting parallel group randomised trials. Int J Surg.

[18] Ottenheijm RP, Jansen MJ, Staal JB, van den Bruel A, Weijers RE, de Bie RA (2010). Accuracy of diagnostic ultrasound in patients with suspected subacromial disorders: a systematic review and meta-analysis. Arch Phys Med Rehabil.

[19] Chen MJ, Lew HL, Hsu TC, Tsai WC, Lin WC, Tang SF (2006). Ultrasound-guided shoulder injections in the treatment of subacromial bursitis. Am J Phys Med Rehabil.

[20] Naredo E, Cabero F, Beneyto P, Cruz A, Mondejar B, Uson J (2004). A randomized comparative study of short term response to blind injection versus sonographic-guided injection of local corticosteroids in patients with painful shoulder. J Rheumatol.

[21] Sibbitt WL, Peisajovich A, Michael AA, Park KS, Sibbitt RR, Band PA (2009). Does sonographic needle guidance affect the clinical outcome of intraarticular injections?. J Rheumatol.

[22] Toigo M, Boutellier U (2006). New fundamental resistance exercise determinants of molecular and cellular muscle adaptations. Eur J Appl Physiol.

[23] Cools AM, Struyf F, De MK, Maenhout A, Castelein B, Cagnie B (2014). Rehabilitation of scapular dyskinesis: from the office worker to the elite overhead athlete. Br J Sports Med.

[24] Hawkins RJ, Kennedy JC (1980). Impingement syndrome in athletes. Am J Sports Med.

[25] van Holsbeeck M, Strouse PJ (1993). Sonography of the shoulder: evaluation of the subacromial-subdeltoid bursa. AJR Am J Roentgenol.

[26] Kolber MJ, Beekhuizen K, Cheng MS, Fiebert IM (2007). The reliability of hand-held dynamometry in measuring isometric strength of the shoulder internal and external rotator musculature using a stabilization device. Physiother Theory Pract.

[27] Thorborg K, Petersen J, Magnusson SP, Holmich P (2009). Clinical assessment of hip strength using a hand-held dynamometer is reliable. Scand J Med Sci Sports.

[28] Roy JS, MacDermid JC, Orton B, Tran T, Faber KJ, Drosdowech D (2009). The concurrent validity of a hand-held versus a stationary dynamometer in testing isometric shoulder strength. J Hand Ther.

[29] van der Heijden GJ, Leffers P, Bouter LM (2000). Shoulder disability questionnaire design and responsiveness of a functional status measure. J Clin Epidemiol.

[30] Munn J, Herbert RD, Gandevia SC (2004). Contralateral effects of unilateral resistance training: a meta-analysis. J Appl Physiol.

[31] van der Windt DA, van der Heijden GJ, de Winter AF, Koes BW, Deville W, Bouter LM (1998). The responsiveness of the Shoulder Disability Questionnaire. Ann Rheum Dis.

[32] Moher D, Hopewell S, Schulz KF, Montori V, Gotzsche PC, Devereaux PJ (2010). CONSORT 2010 Explanation and Elaboration: Updated guidelines for reporting parallel group randomised trials. J Clin Epidemiol.

[33] Holmgren T, Bjornsson HH, Oberg B, Adolfsson L, Johansson K (2012). Effect of specific exercise strategy on need for surgery in patients with subacromial impingement syndrome: randomised controlled study. BMJ..

[34] Bennell K, Wee E, Coburn S, Green S, Harris A, Staples M (2010). Efficacy of standardised manual therapy and home exercise programme for chronic rotator cuff disease: randomised placebo controlled trial. BMJ..

[35] Crawshaw DP, Helliwell PS, Hensor EM, Hay EM, Aldous SJ, Conaghan PG (2010). Exercise therapy after corticosteroid injection for moderate to severe shoulder pain: large pragmatic randomised trial. BMJ..

[36] Hay EM, Thomas E, Paterson SM, Dziedzic K, Croft PR (2003). A pragmatic randomised controlled trial of local corticosteroid injection and physiotherapy for the treatment of new episodes of unilateral shoulder pain in primary care. Ann Rheum Dis.

[37] Jowett S, Crawshaw DP, Helliwell PS, Hensor EM, Hay EM, Conaghan PG (2013). Cost-effectiveness of exercise therapy after corticosteroid injection for moderate to severe shoulder pain due to subacromial impingement syndrome: a trial-based analysis. Rheumatology (Oxford).

[38] Hegedus EJ, Goode A, Campbell S, Morin A, Tamaddoni M, Moorman CT (2008). Physical examination tests of the shoulder: a systematic review with meta-analysis of individual tests. Br J Sports Med.

[39] Iagnocco A, Filippucci E, Meenagh G, Delle SA, Riente L, Bombardieri S (2006). Ultrasound imaging for the rheumatologist. I. Ultrasonography of the shoulder. Clin Exp Rheumatol.

[40] Naredo E, Aguado P, De Miguel E, Uson J, Mayordomo L, Gijon-Baños J (2002). Painful shoulder: comparison of physical examination and ultrasonographic findings. Ann Rheum Dis.

[41] Bennell KL, Hunt MA, Wrigley TV, Hunter DJ, McManus FJ, Hodges PW (2010). Hip strengthening reduces symptoms but not knee load in people with medial knee osteoarthritis and varus malalignment: a randomised controlled trial. Osteoarthritis Cartilage.

