# Supplementary material for: Exercise therapy after ultrasound-guided corticosteroid injections in patients with subacromial pain syndrome: a randomized controlled trial
Source: Arthritis Res Ther. 2016 Jun 4;18:129. doi: 10.1186/s13075-016-1002-5 (PMC4893247; doi:10.1186/s13075-016-1002-5)
Supplement: Additional file 1: — The exercise intervention. The file describes the exercise intervention in detail. (DOC 35 kb) [file 13075_2016_1002_MOESM1_ESM.doc]

**The exercise intervention**

Both groups performed the exact same exercise program. The intervention group exercised their involved shoulder, whereas the control group only exercised their non-involved shoulder, except for exercise 3, which was bilateral.

The exercise program consisted of 10 weeks training with three sessions per week, one supervised by a physiotherapist, and two at home without supervision. 24-hour rests between sessions were recommended.

The exercise program was divided into two phases:

**Phase 1** (2 weeks) **– Muscle control of the scapula:**

**Exercise 1: figure of eight**

Position: Place your body in walking position with the upper body bent slightly forward. The leg on the intervention side is placed backwards. The opposite arm should be leaning gently on a table of appropriate height (shoulder relaxed). The intervention arm is hanging, fingers pointing towards the ground.

Action: The scapula orientation exercise is initially used to posterior tilt and upward rotate the scapula, while increasing scapular muscle activity, by asking the patient to move the scapula backwards and away from an indication of the coracoid process, the arm is moved in very slowly in eight number shapes. When this movement can be performed with proper technique, extra weight of unknown relative load (Repetition Maximum) is added to the arm, by holding it with the hand.

Exercise dose: 20 repetitions, 10 in each direction.

**Exercise 2: Unloaded abduction**

Position: Standing upright with both arms along the side.

Action: The scapula orientation exercise is initially used to posterior tilt and upward rotates the scapula, while increasing scapular muscle activity, by asking the patient to move the scapula backwards and away from an indication of the coracoid process. The arm is then abducted to the horizontal level and back in the plane of the scapula to facilitate scapula co-contraction during open-kinetic chain arm movement.

Exercise dose: 10 repetitions.

**Exercise 3: Wall push-up**

Position: Standing upright and facing the wall, with the arms stretch and touching the wall with the palm of the hands. The hands are placed under shoulder level. The body should be in an inclined position.

Action: The scapula orientation exercise is initially used to posterior tilt and upward rotates the scapula, while increasing scapular muscle activity, by asking the patient to move the scapula backwards and away from an indication of the coracoid process. Slowly and controlled the arms are flexed so that the body approaches the wall. Try to get as close as possible to the wall, before slowly and controlled extending the arms to regain the starting position. This is done to facilitate scapula co-contraction during open-kinetic chain arm movement. The upper-body should be fixated, so that motion occurs mainly in the joints of the arms and ankles.

Exercise dose: 10 repetitions.

**Phase 2** (8 weeks) **– Progressive strength training of the rotator cuff muscles:**

The phase 1 exercises were used as warm up, to facilitate awareness of scapula control during the subsequent 4 strength training exercises targeting the rotator cuff muscles. The focus was to increase muscle strength of the shoulder: abductors, external and internal rotators. A classic linear progression model was used with loads of 20 RM (Repetition-Maximum) at week 3 and 4, 15 RM at week 5 and 6, 12 RM at week 7 and 8, and 10 RM at week 9 and 10. All 3 sets for each strength training exercise were performed until contraction failure (muscular exhaustion). As soon as the patients were able to perform more contractions than that corresponding to the intended relative load (RM), additional external resistance was added by increasing the rubber band tension, or changing to a thicker rubber band. If the exercises caused shoulder pain of more than 50mm VAS, which did not subside immediately, loading was reduced so that the exercise could be performed to contraction failure but with less pain than 50 mm VAS. Repetitions and loads were recorded in a training diary for every training session. Shoulder pain at rest and during movement were recorded before and after each training session with the physiotherapist.

**Exercise 4: Abduction**

Position: Standing with the theraband tight around the intervention hand and the other end placed underneath the feet. Tension in the theraband from the beginning of movement is required.

Action: The scapula orientation exercise is initially used to posterior tilt and upward rotates the scapula, while increasing scapular muscle activity, by asking the patient to move the scapula backwards and away from an indication of the coracoid process. The upper-body is fixated, and the arm is abducted to horizontal level and back to starting position. The duration of the contraction modes is 3 seconds (concentric) 3 seconds (eccentric), and 0 seconds (isometric, no pauses at top or bottom).

Exercise dose: 3 sets per session with 60-second rest between sets.

**Exercise 5: External rotation**

Position: Standing with the opposite site of the intervention arm to a door. The theraband is fastened to the door handle and around the hand. The theraband should go in a horizontal line from handle to the stomach. The elbow is placed in a 90^0^ flexion with a cushion under the upper arm to create a small abduction of the shoulder joint.

Action: The scapula orientation exercise is initially used to posterior tilt and upward rotates the scapula, while increasing scapular muscle activity, by asking the patient to move the scapula backwards and away from an indication of the coracoid process. The upper-body is fixated, and the arm is moved slowly away from the door until full external rotation or pain sensation is reached, then back to starting point. The elbow is kept close to the body. The duration of the contraction modes is 3 seconds (concentric) 3 seconds (eccentric), and 0 seconds (isometric, no pauses between changes of movement direction).

Exercise dose: 3 sets per session with 60-second rest between each set.

**Exercise 6: Internal rotation**

Position: Standing with the intervention arm towards the door, theraband fast at the door handle and hand. Bend the elbow to 90^0^ and rotate the arm as far external as possible. There should be tension in the theraband before movement is initiated. A cushion is placed between the upper arm and side to create a small abduction in the shoulder joint.

Action: The scapula orientation exercise is initially used to posterior tilt and upward rotates the scapula, while increasing scapular muscle activity, by asking the patient to move the scapula backwards and away from an indication of the coracoid process. The upper-body is fixated, and the arm is moved slowly towards the stomach; keeping the elbow at the same place during the movement. The duration of the contraction modes is 3 seconds (concentric) 3 seconds (eccentric), and 0 seconds (isometric, no pauses between changes of movement direction).

Exercise dose: 3 sets per session with 60-second rest between each set.

**Exercise 7: low rows**

Position: Standing in walking position (as exercise 1). Fasten the theraband under the foot opposite the intervention side and in the intervention hand. The theraband should be tight from the beginning of movement.

Action: The scapula orientation exercise is initially used to posterior tilt and upward rotates the scapula, while increasing scapular muscle activity, by asking the patient to move the scapula backwards and away from an indication of the coracoid process. The upper-body is fixated, and the arm is pulled back by extending the shoulder joint and flexing the elbow joint, keeping the arm close to the trunk. The duration of the contraction modes is 3 seconds (concentric) 3 seconds (eccentric), and 0 seconds (isometric, no pauses between changes of movement direction).

Exercise dose: 3 sets per session with 60-second rest between.
